# Using dried blood spot for the detection of HBsAg and anti-HCV antibodies in Cameroon

**DOI:** 10.1186/s13104-018-3931-3

**Published:** 2018-11-16

**Authors:** Sebastien Kenmoe, Paul Alain Ngoupo Tagnouokam, Cyprien Kengne Nde, Ghislaine Flore Mella-Tamko, Richard Njouom

**Affiliations:** 1 Virology Department, Centre Pasteur du Cameroun, 451 Rue 2005, Yaoundé 2, P.O.Box 1274, Yaounde, Cameroon; 2 National AIDS Control Committee, Yaounde, Cameroon

**Keywords:** Dried blood spot, Diagnosis, Hepatitis B, Hepatitis C, Cameroon

## Abstract

**Objective:**

Dried blood spots (DBS) offer multiple benefits for collecting, storing and shipping whole blood samples. Our objective was to compare, for the first time in Africa, the performance of DBS with respect to plasma in the detection of Hepatitis B surface antigen (HBsAg) and antibodies to Hepatitis C Virus (anti-HCV) using Architect, Abbott Diagnostics.

**Results:**

DBS had a sensitivity of 99%, a specificity of 100%, a positive predictive value of 99%, a negative predictive value of 100% and a kappa index of 0.99 for the detection of HBsAg. For anti-HCV detection, the sensitivity, specificity, positive predictive value, negative predictive value and kappa index were 99%, 98%, 98%, 99%, and 0.97, respectively. This study confirms that DBS may be a reliable alternative specimen type for HBV and HCV diagnosis.

## Introduction

According to the WHO report published in 2017, more than 250 million and about 70 million persons are chronically infected with Hepatitis B virus (HBV) and Hepatitis C virus (HCV), respectively [1]. Sub-Saharan Africa is the region with the highest burden of HBV infection (prevalence > 8%) [2]. Data from a national survey conducted in Cameroon in 2011 showed a prevalence of 12% and 1% of HBV and HCV, respectively in the general population  [3]. Diagnosis remains the cornerstone for therapeutic actions as well as for preventive actions for hepatitis viral infection. In low-income countries, the lack of diagnostic infrastructure in rural and peripheral areas represents a major barrier to access to viral hepatitis screening  [4]. The detection of viral hepatitis diagnosis is typically based on venous blood. However, dried blood spot (DBS) has been shown to be an effective alternative to serum or plasma in people of all ages [5]. According to WHO guidelines, priority actions for HBV and HCV testing include the use of DBS in regions that lack infrastructure or expertise for venous blood collection and for vulnerable populations [6]. However, the performance and feasibility of DBS use for the diagnosis Hepatitis C and B have not yet been described in many low-income countries in Sub-Saharan Africa including Cameroon. The present work was designed to evaluate the diagnostic accuracy of DBS for Hepatitis B surface antigen (HBsAg) and HCV antibodies (anti-HCV) detection.

## Main text

### Materials and methods

This cross-sectional study included 400 paired plasma and DBS samples (100 HBsAg positive, 100 anti-HCV positive and 100 HBsAg negative and, 100 anti-HCV negative) collected at the Centre Pasteur of Cameroon (CPC). The samples were obtained from patients received at CPC as part of the routine HBsAg and anti-HCV screening activities and no additional parameter was assessed. Paired plasma and DBS samples were prospectively prepared for all study samples. A 4 mL whole blood was collected from each patient and five spots of 50 µL were prepared on Whatman 903 protein saver card (Whatman, GE Healthcare, NJ). The blood samples were further centrifuged for 10 min at 2500 rpm and plasma was immediately tested. For DBS, two dishes were cut and transferred into 1.5 mL tube. Elution of DBS was further performed with 500 μL of PBS (pH 7.2) followed by overnight incubation at + 4 °C. The next day, the eluates were subjected to centrifugation at 10,000 rcf for 5 min, and the supernatant was used for HBsAg and anti-HCV screening.

Plasma and DBS were tested using Architect HBsAg Qualitative II and Architect Anti-HCV assays (Abbott Diagnostics, Wiesbaden, Germany), according to the manufacturer’s instructions. Samples with cut-off values (S/Co) < 1 were considered negative and those with S/Co ≥ 1 were considered positive for HBsAg and anti-HCV, as recommended by the manufacturer.

The results obtained with plasma were used as reference to assess sensitivity, specificity, positive predictive value, and negative predictive value. To obtain the best sensitivity and specificity, we estimated the optimal threshold value based on the sensitivity and specificity curves with respect to threshold values. The concordance between the results obtained from analysis of plasma and DBS was evaluated using the kappa index. P-values < 0.05 was considered statistically significant. Statistical analyses were performed using R software version 3.4.1.

### Results

Considering the plasma results as a reference, all the 100 HBV positive samples were confirmed for HBsAg testing using DBS. Fourteen HBsAg negative plasma samples were weakly positive (mean ratio 1.2 ± 0.07) on DBS. These samples were re-tested as recommended by the manufacturer, and 13 of them became negative. The first results were therefore considered as false positives and, only 1 false positive result was finally obtained with DBS. The sensitivity, specificity, positive predictive value and negative predictive value were 100%, 99%, 99% and 100% respectively (Table 1). Moreover, an excellent agreement (kappa = 0.99) was obtained between plasma and DBS results.

**Table 1:** Sensitivity, specificity, positive predictive value, negative predictive value, and kappa index for the detection of HBsAg and anti-HCV on DBS compared to plasma

	HBsAg			Anti-HCV		
	Value	95% CI	p-value	Value	95% CI	p-value
Se	100	[96.3–100]	1	99	[94.5–99.9]	< 0.01
Sp	99	[94.5–99.9]	< 0.01	98	[92.9–99.7]	< 0.01
PPV	99.0	[94.6–99.9]	< 0.01	98	[93.0–99.7]	< 0.01
NPV	100	[96.3–100]	1	99	[94.5–99.9]	< 0.01
Kappa index	0.99	[0.96–1]	–	0.97	[0.93–1]	–

Se, Sp, PPV, and NPV values for anti-HCV were obtained with adjusted cut-off value of 0.73

*Se* sensitivity, *Sp* specificity, *PPV* positive predictive value, *NPV* negative predictive value

The results of the 100 anti-HCV negative plasma samples were similar on DBS. Twenty-three of the 100 anti-HCV positive plasma samples were negative on DBS. These 23 false negatives were tested a second time and the same results were obtained.

**Fig. 1:**
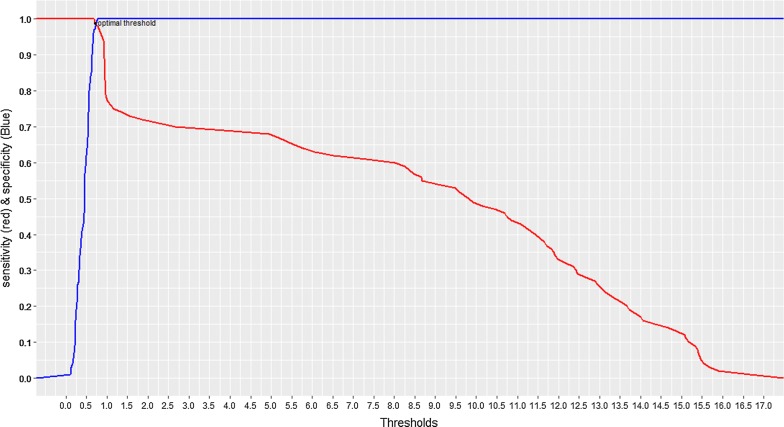
Plot of sensitivity and specificity versus signal-to-cutoff values of hepatitis C virus testing using DBS in Cameroon. Optimal cutoff value: 0.73

A new cut-off value of 0.73 was set for HCV results based on the sensitivity and specificity curves with respect to threshold values (Fig. 1). Five false negative results were maintained after adjusting the S/Co value from 1 to 0.73. Thus, a specificity of 98%, a sensitivity of 99%, a positive predictive value of 98% and a negative predictive value of 99% were obtained for anti-HCV detection using DBS. The Kappa index (0.97) shows an excellent agreement between the results obtained with plasma and DBS samples.

### Discussion

In this work, we evaluated the performance HBV and HCV screening using DBS. Similar to previous study using the same method (Architect, Abbott diagnostics), for the detection of HBsAg, a perfect sensitivity (100%) and a good specificity (99%) were obtained with the DBS, and the kappa index (0.99) also indicated excellent agreement between plasma and DBS [6–8]. Although we applied a lower blood volume (50 μL) on DBS than Ross et al. (100 μL) and Mössner et al. (75 μL), used a lower elution solution (500 μL vs 1000 μL), and used a venous blood compared to the capillary blood used by Mössner et al. Our results reinforce the good performance and confirm the robustness of the use of DBS in HBV screening [7, 8]. However, one should be cautious because Forbi et al. obtained a low sensitivity of 78.6% and a low specificity of 88.6% for detection of anti-HCV on DBS when using a lower blood volume (25 μL) and a variable threshold value depending on the negative control in a study in Nigeria [9]. This variability of the results according to the studies thus justifies the performance evaluation of the DBS in the diagnosis of HBV or HCV for the implementation of the useful recommendations.

For the anti-HCV test, we obtained 23 false negatives and 100% specificity by applying the threshold value recommended by the manufacturer of the kit (1). As reported previously [10], our data showed that all these 23 false negative samples had significantly lower mean titers than those obtained on plasma (DBS 0.5 ± 0.2 vs plasma 3.4 ± 1.9). Compared to previous studies that had better sensitivities, the low sensitivity in the detection of HCV in this study could be explained by the low blood volume deposited on DBS [7, 8]. Other authors have indeed suggested that low blood volumes (< 50 μL) could significantly reduce the detection sensitivity of HCV [11, 12].

After adjusting to the best cut-off value as proposed by other authors for the detection of anti-HCV on DBS [10, 12–14], we obtain 98% specificity and 99% sensitivity, which is consistent with previous studies [7, 8]. Therefore our results suggest that a cut-off value of 0.73 could be used for testing anti-HCV on DBS in our context.

The low availability of specialized laboratories in remote areas is one of the major barriers to large scale testing for HBV and HCV in Africa. DBS could therefore be an alternative, simple and less expensive method to facilitate access to HBV and HCV screening especially for people living in peripheral regions. In this context, it is essential to assess the stability of DBS samples in different shipment temperatures and durations. However, Mohamed et al. have previously reported that there was no significant variation in the HBsAg DBS positive samples stored at room temperature between 1 and 14 days, indicating that DBS could have a good stability at room temperature for a long period [15].

In summary, DBS is a useful, alternative, and simple method in HBV and HCV screening. Using DBS will greatly improve accessibility to HBV and HCV screening and to treatment for populations living in remote areas with limited infrastructure for HBV and HCV diagnosis.

## Limitations

Although the present study is the first in Africa to evaluate the performance of DBS with the widely used method for hepatitis B and hepatitis C diagnosis (Architect, Abbott Diagnostics), we did not assess the impact of transport and storage conditions on DBS.
